# CARE-RECEIVING CHARACTERISTICS AMONG OLDER ADULTS WITH DIFFERENT MENTAL–SOMATIC MULTIMORBIDITY COMBINATIONS

**DOI:** 10.1093/geroni/igad104.2648

**Published:** 2023-12-21

**Authors:** Mona Liu, Corey Nagel, Siting Chen, Heather Allore, Jeffrey Kaye, Ana Quiñones

**Affiliations:** Oregon Health & Science University, Portland, Oregon, United States; University of Arkansas for Medical Sciences, Little Rock, Arkansas, United States; Oregon Health & Science University, Portland, Oregon, United States; Yale University, New Haven, Connecticut, United States; Oregon Health & Science University, Portland, Oregon, United States; Oregon Health & Science University, Portland, Oregon, United States

## Abstract

Older adults with distinct multimorbidity combinations require different care with varying intensities. This study aimed to examine sociodemographic, health, and care-receiving characteristics among older adults with varying multimorbidity patterns. A total of 4,719 participants from the National Health and Aging Trends Study in 2017 were included, and five clinically-informed multimorbidity categories were assessed: 169 (3.58%) had no conditions, 3031 (64.23%) had somatic conditions only, 347 (7.35%) had depression without cognitive impairment (depression group), 852 (18.05%) had cognitive impairment without depression (cognitive impairment group), and 320 (6.78%) had both depression and cognitive impairment. Compared with the depression group and cognitive impairment group, those with both depression and cognitive impairment were significantly older, more likely to be non-Hispanic Black or Hispanic, widowed, U.S. born, Medicaid recipients, underweight BMI, had lower levels of education and more functional limitations. Compared to the depression group, those with both depression and cognitive impairment had lower self-rated health, less likely to be a smoker, received 118 more hours of care per month on average with more caregivers in ADLs, banking, and money management. Compared with the cognitive impairment group, those with both depression and cognitive impairment had more somatic conditions, received an average of 70 more hours of care per month with more caregivers in ADLs. Results highlighted the substantially greater caregiving hours and intensities pertaining to the combination of depression and/or cognitive impairment, and suggested the potential of addressing mental health among older adults with or without dementia to improve their own and caregivers’ health outcomes.

